# Genetic Determinants Linked to MDR/XDR in *Pseudomonas aeruginosa* Strains from a Mexican Tertiary Hospital

**DOI:** 10.3390/pathogens15010100

**Published:** 2026-01-17

**Authors:** Liliana Nicolas-Sayago, Miguel Ángel Loyola-Cruz, Yesseny Vásquez-Martínez, Marcelo Cortez-San Martín, Laura Margarita Márquez-Valdelamar, Clemente Cruz-Cruz, Emilio Mariano Durán-Manuel, Mireya Ruíz-Valdés, Claudia Camelia Calzada-Mendoza, Araceli Rojas-Bernabé, María Concepción Tamayo-Ordóñez, Yahaira de Jesús Tamayo-Ordóñez, Julio César Castañeda-Ortega, Briceida López-Martínez, Benito Hernández-Castellanos, Daniela Moreno-Torres, Graciela Castro-Escarpulli, Juan Manuel Bello-López

**Affiliations:** 1 Hospital Juárez de México, Mexico City 07760, Mexico; 2 Escuela Nacional de Ciencias Biológicas, Instituto Politécnico Nacional, Mexico City 11340, Mexico; 3 Escuela de Medicina, Facultad de Ciencias Médicas, Universidad de Santiago de Chile, Santiago 9170022, Chile; 4 Laboratorio de Virología Molecular y Control de Patógenos, Facultad de Química y Biología, Universidad de Santiago de Chile, Santiago 9170022, Chile; 5 Laboratorio de Secuenciación Genómica, Laboratorio Nacional de Biodiversidad (LaNaBio), Instituto de Biología, Universidad Nacional Autónoma de México (UNAM), Mexico City 04510, Mexico; 6 Sección de Estudios de Posgrado e Investigación, Escuela Superior de Medicina, Instituto Politécnico Nacional, Mexico City 11340, Mexico; 7 Facultad de Ciencias Químicas, Universidad Autónoma de Coahuila, Saltillo 25280, Mexico; 8 Centro de Biotecnología Genómica, Instituto Politécnico Nacional, Reynosa 88710, Mexico; 9 Facultad de Biología, Universidad Veracruzana, Xalapa 91090, Mexico

**Keywords:** *Pseudomonas aeruginosa*, β-lactamases, *oprD* mutation, MDR/XDR phenotypes

## Abstract

Background: *Pseudomonas aeruginosa* is one of the leading agents causing healthcare-associated infections (HAIs) due to its intrinsic resistance, its capacity to acquire resistance mechanisms, and its persistence in hospital environments. In Mexico, it ranks among the most frequently reported pathogens in national surveillance systems. The aim of this study was to characterize antimicrobial resistance profiles and the genetic determinants associated with MDR/XDR phenotypes in *P. aeruginosa* strains from HAIs at Hospital Juárez de México (HJM). Methods: Sixty-three strains from patients with HAIs were analyzed. Identification was confirmed by *16S rRNA* gene sequencing. Antimicrobial susceptibility testing followed CLSI guidelines. MDR/XDR phenotypes were classified according to the Latin American consensus for categorizing MDR, XDR, and PDR pathogens. Screening for resistance mechanisms was carried out by PCR for the main β-lactamases circulating at HJM. Finally, mutations in the *oprD* gene were detected in imipenem-resistant isolates through amino acid sequence alignment. Results: Resistant phenotypes allowed the identification of MDR and XDR profiles. Only the metallo-β-lactamase *bla_VIM_* was detected. Analysis of *oprD* porin sequences revealed recurrent mutations (S103T, T115K, L170F, G186P, and T189V) associated with imipenem resistance. Conclusions: In *P. aeruginosa*, the presence of *bla_VIM_* and structural alterations in OprD confirms the multifactorial nature of carbapenem resistance. These findings underscore the need to strengthen microbiological surveillance programs and antimicrobial stewardship strategies to mitigate the impact and spread of MDR/XDR isolates.

## 1. Introduction

Healthcare-associated infections (HAIs) constitute one of the major public health challenges worldwide due to their morbidity, mortality, and economic burden, particularly among critically ill patients in intensive care units (ICUs) and those requiring invasive procedures such as mechanical ventilation, catheters, or urinary devices [1,2]. Within this context, *Pseudomonas aeruginosa*, a member of the ESKAPE group (*Enterococcus faecium*, *Staphylococcus aureus*, *Klebsiella pneumoniae*, *Acinetobacter baumannii*, *Pseudomonas aeruginosa*, and *Enterobacter* spp.), stands out as an opportunistic pathogen classified by the WHO as a high-priority organism due to its carbapenem resistance and its ability to cause a wide range of infections in immunocompromised patients [3,4,5]. This Gram-negative, non-fermenting bacillus exhibits remarkable genomic plasticity, can persist in humid environments, form mature biofilms, and colonize inert surfaces—including invasive medical devices [6,7,8]. Its metabolic versatility, combined with an extensive repertoire of virulence factors such as adhesins, polar flagella, pili, secretion systems, and exoenzymes, facilitates colonization and infection of multiple anatomical sites, leading to ventilator-associated pneumonia (VAP), catheter-related bloodstream infections (CLABSI), catheter-associated urinary tract infections (CAUTI), and surgical site infections (SSI) [9,10,11].

Because of this, *P. aeruginosa* is considered a key ESKAPE pathogen, capable of “escaping” the action of antimicrobials and responsible for a substantial proportion of HAIs worldwide [12,13,14]. Concern over this bacterium and other ESKAPE pathogens has intensified due to increasing carbapenem resistance (meropenem and imipenem), which limits therapeutic options and underscores the need for robust microbiological surveillance to guide clinical decisions [15,16]. The clinical relevance of *P. aeruginosa* is rooted in the combined effect of virulence and resistance. The type III secretion system, for example, acts as a “molecular syringe,” injecting effectors into host cells and disrupting defense mechanisms. Moreover, biofilm formation not only protects against immune response but also limits antibiotic penetration, promoting chronic infections and relapses [17,18].

Antimicrobial resistance in *P. aeruginosa* is driven by reduced outer membrane permeability, basal production of chromosomal AmpC β-lactamases, efflux pump overexpression, acquisition of extended-spectrum β-lactamases (ESBLs), and, notably, serine and metallo-β-lactamases with carbapenem-hydrolyzing activity [19,20,21]. Another key mechanism of imipenem resistance involves alterations in the OprD porin, the main entry channel for imipenem and small basic peptides. Mutations—especially in external loops such as L2 and L3—or loss of the porin reduce permeability and lead to resistance even in the absence of carbapenemase genes [22,23,24].

The increasing frequency of resistant phenotypes prompted the development of consensus definitions to categorize *P. aeruginosa* isolates based on resistance spectrum: multidrug-resistant (MDR) isolates are resistant to at least one agent in three or more antimicrobial categories; extensively drug-resistant (XDR) isolates retain susceptibility to only one or two antimicrobial categories; and pandrug-resistant (PDR) isolates are resistant to all tested agents [25,26,27]. Complementary definitions, such as the “difficult-to-treat resistance” (DTR) category for Gram-negative pathogens, provide a unified terminology to integrate phenotypic and genotypic data, facilitating clinical–microbiological correlations and antimicrobial decision-making [28,29,30].

MDR and XDR *P. aeruginosa* infections have been associated with prolonged hospital stays, increased costs, the need for toxic or less accessible antibiotic combinations, and poorer outcomes, especially in critically ill patients [31]. In Mexico, national HAI surveillance has consistently documented *P. aeruginosa* among the most frequently isolated pathogens in VAP, CLABSI, and CAUTI. At HJM, we have identified *P. aeruginosa* as an important pathogen in critical areas where environmental persistence and antimicrobial resistance complicate control measures and treatment. Characterizing resistance mechanisms at the local level is crucial for treatment success and informs the use of new β-lactam/β-lactamase inhibitor combinations such as cefiderocol, supporting antimicrobial stewardship and prevention strategies [32]. The aim of this study was to characterize carbapenem resistance mechanisms in clinical isolates of *P. aeruginosa* by integrating phenotypic and genotypic data, focusing on the detection of serine and metallo-β-lactamases and mutations in the *oprD* gene. Implications for nosocomial spread of MDR and XDR *P. aeruginosa* isolates lacking carbapenemase production are discussed.

## 2. Materials and Methods

### 2.1. Pseudomonas Aeruginosa Strains and Biochemical Identification

A total of 63 *P. aeruginosa* strains were isolated from non-duplicate patients with confirmed HAIs (and without previous antimicrobial treatment) at HJM during 2021 and 2022. Isolates were obtained from four clinical sources—bronchoaspirate, urine, blood, and soft tissue—corresponding to VAP, CAUTI, CLABSI, and SSI, respectively. Bacterial isolation followed classical bacteriological culture protocols on selective and differential media for Gram-negative non-fermenting bacilli (Mac Conkey). Identification at the genus and species levels was performed using the automated Vitek^®^ 2-XL system (bioMérieux, Durham, NC, USA) following the manufacturer’s instructions.

### 2.2. Genetic Confirmation of Pseudomonas aeruginosa by 16S rRNA Sequencing

For molecular analyses, total genomic DNA from each strain was extracted and purified using the DNeasy Blood & Tissue Kit (QIAGEN, Venlo, The Netherlands). DNA integrity was verified on 0.8% agarose gels and used as a template for endpoint PCR assays. Amplifications were performed in a T100™ Thermal Cycler in a final volume of 50 μL (Bio-Rad, Hercules, CA, USA). The full-length *16S rRNA* gene was amplified using universal primers 27F (5′-AGAGTTTGATCMTGGCTCAG-3′) and 1492R (5′-TACGGYTACCTTGTTACGACTT-3′) (0.3 μm), according to the conditions described by DeSantis et al. [33]. Amplicons were resolved on 1.5% agarose gels using 1× TBE buffer, purified, and sequenced at the Institute of Biology of the Universidad Nacional Autónoma de México (UNAM) using an ABI 3730xL DNA Analyzer (Applied Biosystems, Foster City, CA, USA). Sequences were compared against the GenBank nucleotide database using the BLAST algorithm (NCBI BLAST+, web interface; BLAST+ 2.x), with coverage ≥ 95%, identity ≥ 98.7% and e-value 0.0 as acceptance thresholds [34,35].

### 2.3. Antimicrobial Resistance Profiles

Antimicrobial susceptibility profiles were determined according to CLSI (2024) guidelines for *P. aeruginosa* [36]. Colistin susceptibility was evaluated by broth macrodilution in cation-adjusted Mueller–Hinton broth at concentrations of 1, 2, and 4 µg/mL. *Pseudomonas aeruginosa* ATCC 27853 and *Escherichia coli* ATCC 25922 served as quality controls. Classification of MDR, XDR, and PDR phenotypes followed the Latin American consensus for resistance taxonomy [37].

### 2.4. Confirmation of Carbapenemase Production

Carbapenem-resistant isolates were evaluated using the modified Carbapenem Inactivation Method (mCIM) according to Pierce et al. [38]. Briefly, two 1 µL loopfuls of overnight culture were suspended in 2 mL of trypticase soy broth (TSB), and a 10 µg meropenem (MEM) disk (Becton Dickinson and Company, Sparks, MD, USA) was immersed in the suspension and incubated at 37 °C for 4 h. Mueller–Hinton agar plates previously inoculated with a lawn of *E. coli* ATCC 25922 (0.5 McFarland) were used as indicator plates. After incubation, inhibition zones were measured following disk diffusion criteria according to CLSI. *Klebsiella pneumoniae* strain carrying *bla_NDM-1_* gene [39] was used as positive control and *E. coli* ATCC 25922 as a negative control.

### 2.5. β-Lactam Resistance Gene Screening

Endpoint PCR assays were used to detect the metallo-β-lactamase genes *bla_NDM_*, *bla_VIM_*, and *bla_IMP_*, and the serine β-lactamase gene *bla_KPC_*. Amplifications followed conditions reported by Dallenne et al. [40] and Nordmann et al. [41]. Amplicons were separated in 2.0% agarose gels in 1× TBE (pH 8.3), visualized under UV light, and compared with appropriate molecular weight markers. Positive controls were obtained from Cortés-Ortíz et al. [39] and Loyola-Cruz et al. [12]. The *bla* gene PCR panel was intentionally restricted to carbapenemase determinants previously documented as circulating at HJM, where *bla_VIM_* has been the main metallo-β-lactamase detected in institutional surveillance. OXA-type carbapenemases were not included due to their low frequency in local clinical cohorts and absence as prevalent circulating mechanisms at HJM.

### 2.6. Detection of Mutations in the oprD Gene in Pseudomonas aeruginosa

To detect mutations in the *oprD* gene and their possible relationship with imipenem resistance in the 27 resistant strains, endpoint PCR reactions were performed to amplify the complete sequence of the gene. Amplification was carried out using the specific primers OprD-F (5′-CGCCGACAAGAAGAACTAGC-3′) and OprD-R (5′-GTCGATTACAGGATCGACAG-3′), as described by Gutiérrez et al. [42]. The amplification conditions were as follows: pre-denaturation at 95 °C for 5 min, followed by 30 cycles consisting of denaturation at 95 °C for 30 s, annealing at 55 °C for 45 s, and extension at 72 °C for 1 min, with a final extension step at 72 °C for 7 min. The expected amplicon size (1412 bp) was confirmed by horizontal electrophoresis on agarose gels. The amplified products were purified and sequenced in both directions (using forward and reverse OprD primers) for subsequent bioinformatic analysis. Sequencing files obtained in .ab1 format were converted to FASTA format, and the sequences were translated into amino acids for further analysis. Sequence alignment was performed at the amino acid level using MEGA X v10.2.6 software and the MUSCLE algorithm. The *OprD* porin of *P. aeruginosa* PAO1, available in the GenBank database under accession number AHY39263.1, was used as the reference sequence.

Mutations were analyzed with emphasis on the regions corresponding to loops 1, 2, and 3 of the porin, where point changes can modify outer membrane permeability and contribute to the imipenem-resistant phenotype [10]. Finally, the positions of affected amino acids, the type of substitution, and the frequency (%) of each mutation were determined. No systematic search for full-gene deletions, insertions, or premature stop codons was performed.

### 2.7. Statistical Analysis

A descriptive and exploratory statistical analysis was performed. Contingency tables compared clinical sources across phenotype categories (MDR, XDR, non-MDR) using Chi-square tests (*df* = 8). Additional contingency analyses were conducted to evaluate potential differences in the distribution of infection sources (VAP, CAUTI, CLABSI, and SSI) between male and female patients, as well as between study years (2021 and 2022). Two-by-two tables evaluated *bla_VIM_* detection (+/−) versus imipenem (IMP) and meropenem (MEM) resistance using Fisher’s exact test (two-sided). Analyses were performed in R v4.3.1, with *p* < 0.05 considered statistically significant.

## 3. Results

### 3.1. Origin of Pseudomonas aeruginosa Isolates

As shown in Table 1, the majority of the 63 *P. aeruginosa* isolates obtained during the study period originated from VAP cases, which remained the predominant HAI in both years analyzed (59.5% in 2021 and 57.7% in 2022). CAUTI accounted for roughly one-quarter of isolates each year, while CLABSI and SSI contributed smaller proportions that remained stable between 2021 and 2022. When considering patient sex, most isolates were recovered from male patients (*n* = 35), although the clinical distribution pattern was similar between sexes: VAP was the principal source of isolation in both groups, and CAUTI, CLABSI, and SSI showed only minor proportional differences.

A similar pattern was observed in 2022, with VAP remaining the most common source of isolation, followed by CAUTI, CLABSI, and SSI. Regarding sex distribution, most isolates were obtained from men (*n* = 35), while 28 cases were from women. In both groups, pulmonary isolates associated with VAP predominated (60.0% in men and 57.1% in women). In contrast, CAUTI isolates were proportionally more frequent in men (25.7%), whereas CLABSI exhibited the same representation in women (17.9%) as CAUTI. SSI-derived isolates were scarce and similarly distributed between sexes (5.7% in men vs. 7.1% in women) (Table 1).

### 3.2. Phenotypic Antimicrobial Resistance Profiles of Pseudomonas aeruginosa Strains

**Figure 1:**
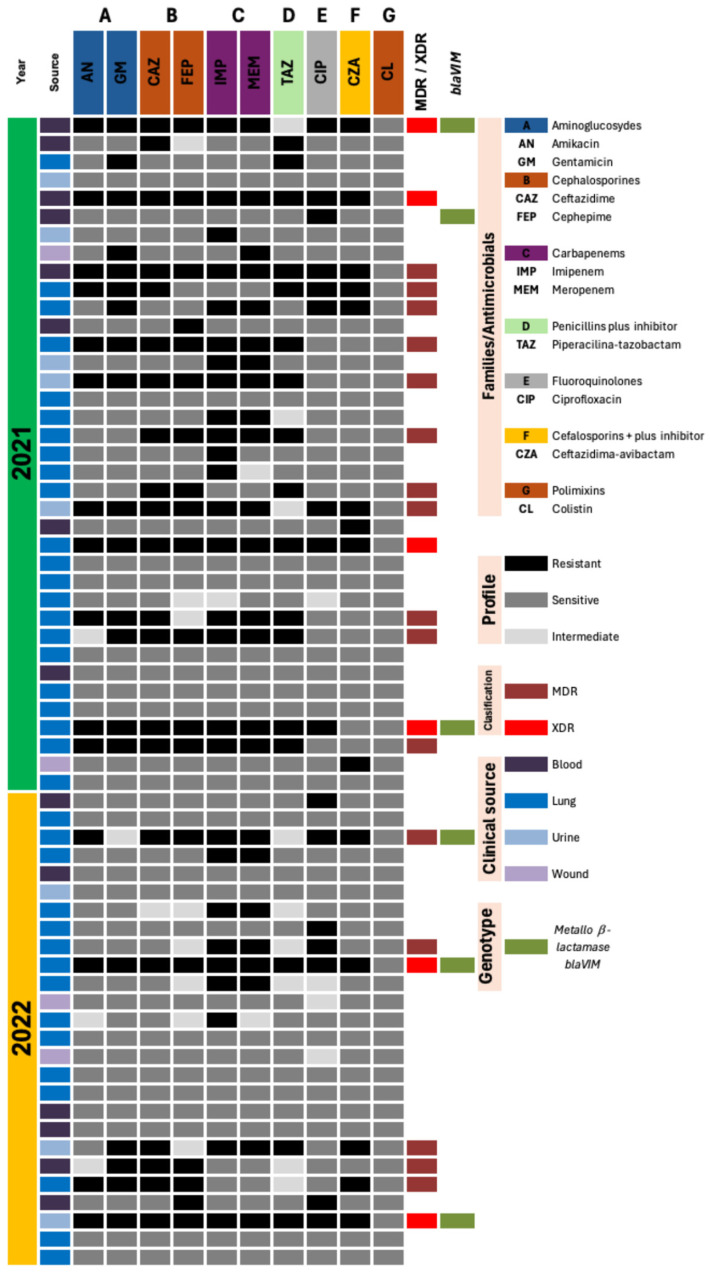
Antimicrobial resistance phenotypes in *Pseudomonas aeruginosa* isolates (2021–2022), showing the distribution across antibiotics, antimicrobial families, MDR/XDR profiles, clinical sources, and detected genotypes. Each row represents one isolate and each column corresponds to an antibiotic or associated variable. Colour legend: Blood = purple, Lung = blue, Urine = light-blue, Wound = lilac; Phenotypic interpretation: Resistant = black, Intermediate = light-gray, Sensitive = gray; Resistance classification: MDR = dark maroon, XDR = red; and Genotype marker: *bla_VIM_*-positive = olive-green.

The antimicrobial resistance profiles of the 63 *P. aeruginosa* isolates revealed broad resistance across multiple antimicrobial families, reflected in the high frequency of MDR (25.4%) and XDR (9.5%) phenotypes observed in the study. Figure 1 shows combined resistance to β-lactams, fourth-generation cephalosporins, carbapenems, fluoroquinolones, and aminoglycosides (represented by black cells). During 2021, a high proportion of isolates showed simultaneous resistance to imipenem, meropenem, cefepime, and ciprofloxacin, with heterogeneous patterns of limited susceptibility to amikacin and complete susceptibility to colistin. Most resistant profiles belonged to the MDR category (*n* = 11), although a subset of XDR isolates was identified (*n* = 4), showing near-complete resistance to the entire antimicrobial panel. These XDR isolates were associated with VAP and CAUTI cases (Figure 1). In 2022, although fewer isolates were obtained, the phenotypic panorama remained essentially unchanged. MDR isolates (*n* = 5) and XDR isolates (*n* = 2) continued to exhibit resistance to multiple antimicrobial classes, with preserved susceptibility to aminoglycosides and colistin. The persistence of MDR and XDR phenotypes across both years could be confirmed their stability and ongoing role as causative agents of HAIs. Despite the notable presence of resistant phenotypes, Figure 1 also reveals a considerable number of isolates with preserved susceptibility to one or more antimicrobial classes (represented by gray cells, 65.1%).

### 3.3. Detection of β-Lactam Resistance Genes

Endpoint PCR analysis in imipenem resistant strains (*n* = 28) revealed that the *bla_VIM_* gene was detected in only six isolates (Figure 1). When comparing genotypic and phenotypic profiles, *bla_VIM_*-positive isolates showed simultaneous resistance to imipenem and meropenem (*n* = 5). One exception was observed in a single isolate that remained phenotypically susceptible to carbapenems despite harboring *bla_VIM_*, representing the only genotype–phenotype discordance identified in this study. Temporally, *bla_VIM_* was detected in both years, with no notable differences in frequency between 2021 and 2022. None of the isolates showed amplification for *bla_IMP_*, *bla_NDM_*, or *bla_KPC_*.

### 3.4. Statistical Analysis

The 63 isolates corresponded to eligible, non-duplicated cases of HAIs from various clinical sources at HJM (VAP, CAUTI, CLABSI, and SSI). When resistance was classified according to the MDR criteria, 16 of the 63 isolates (25.4%) met the criteria for multidrug resistance (MDR), while 6 of 63 (9.5%) were classified as extensively resistant (XDR), and 41 of 63 (65.1%) were non-MDR/non-XDR. No statistically significant association was observed between the clinical source and the resistance category (MDR/XDR/non-MDR) (Chi-square, *df* = 8, *p* = 0.831). Although pulmonary venom was the most frequent source of HAIs in both years (59.5% in 2021 and 57.7% in 2022), this predominance was not statistically significant (Chi-square, *p* = 0.831). Similarly, no significant differences were observed between isolates obtained from male and female patients (*p* > 0.05). Carbapenem resistance (imipenem or meropenem) was present in 27 of the 63 isolates (42.9%). The *bla_VIM_* gene was detected in 6 of the 63 isolates (9.5%); five of the *bla_VIM_*-positive isolates were carbapenem-resistant, with no statistically significant difference compared to imipenem resistance (two-tailed Fisher’s exact test, *p* = 0.152).

### 3.5. Mutations in the oprD Gene and Their Relationship with Imipenem Resistance

**Figure 2:**
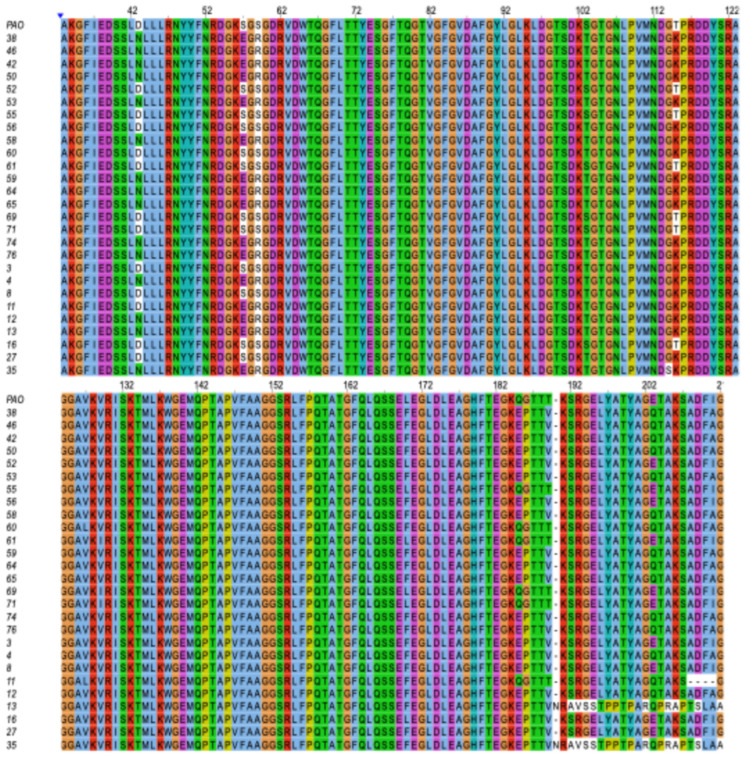
Multiple sequence alignment of the OprD protein from *Pseudomonas aeruginosa* isolates compared with the PAO1 reference sequence. The alignment highlights amino acid substitutions across the analyzed strains, particularly within loops L1, L2, and L3, which are key structural regions associated with imipenem resistance.

Complete sequencing of the *oprD* gene in the 27 *P. aeruginosa* isolates resistant to imipenem allowed retrieval of the full amino acid sequence of the OprD porin and generation of a multiple alignment against the PAO1 reference strain (AHY39263.1). The analysis was intentionally restricted to external loops L1 (positions 43–61), L2 (93–127), and L3 (153–192), which represent the principal permeability-modulating regions associated with impaired imipenem uptake. Notably, a consistent genotype–phenotype pattern was observed within the study set: all *bla_VIM_*-positive isolates (*n* = 6) lacked substitutions in OprD loops, whereas all imipenem-resistant isolates without *bla_VIM_* (*n* = 21) exhibited at least one amino-acid substitution within loops L1–L3 (Figure 2).

In loop 1, three recurrent substitutions were identified: D43N, S57E, and S59R, each present in 15 of the 27 strains (55.6%), reflecting a conserved pattern of alteration in the N-terminal region of the protein. Loop 2 showed the most prevalent mutations, particularly S103T and T115K, both detected in 21 strains (77.8%), in addition to G114S, which was observed in only one strain (3.7%). Loop 3 exhibited the highest diversity of changes, including substitutions such as L170F (21 strains; 77.8%), Q185E (20 strains; 68.7%), G186P (21 strains; 77.8%), T189V (21 strains; 77.8%), and E203Q (17 strains; 63%). Low-frequency mutations (7.1%) were also observed, including K190N, S192A, R193V, G194S, E195S, L196T, Y197P, A198P, and Y200P (Table 2).

The overall analysis showed that most strains accumulated multiple mutations simultaneously, especially recurring combinations within loops 2 and 3. Despite the high frequency of mutations identified in loops 1, 2, and 3 of OprD, the multiple alignment revealed that not all imipenem-resistant strains exhibited substitutions in these regions; several retained a sequence identical to the PAO1 reference in the evaluated sites (*n* = 3). Finally, additional changes were detected outside these domains, although none corresponded to variants previously recognized as functionally relevant; therefore, they were not included in the comparative analysis.

## 4. Discussion

The growing presence of healthcare-associated infections (HAIs) continues to represent a significant challenge for hospital systems, particularly in critical care environments where patients are exposed to invasive devices, multiple comorbidities, and prolonged antimicrobial therapy. In this context, *P. aeruginosa* remains one of the most relevant pathogens due to its remarkable adaptive capacity, genomic plasticity, ability to form mature biofilms, and expression of diverse virulence factors that collectively enhance its persistence and dissemination in healthcare settings [43,44].

According to the national surveillance bulletin in Mexico (RHOVE), *P. aeruginosa* consistently ranks among the primary etiologic agents associated with HAIs, a finding that aligns with what we observed in our hospital during the study period [45]. Importantly, the 63 isolates correspond to all eligible, non-duplicate clinical cases recovered at HJM during the study period, not a selected subset. Indeed, the distribution of isolates demonstrated that VAP was the most frequent HAI, followed by CAUTI, CLABSI, and SSI, consistent with both national and international reports describing a similar pattern [9,10,12]. The results from 2021 and 2022 showed that the respiratory tract remained the main source of *P. aeruginosa* isolates at HJM, which aligns with studies identifying this pathogen as a leading cause of VAP in ICUs. Several reports describe *P. aeruginosa* as a dominant agent in VAP, findings that match ours, in which pulmonary isolates represented the principal clinical manifestation of this pathogen [1,46].

Urinary tract infections (CAUTIs) accounted for 22.2% (*n* = 14) of isolates analyzed, consistent with the epidemiological relevance of urinary infections associated with prolonged catheterization in hospitalized patients [47]. In our study, *P. aeruginosa* emerged as an etiologic agent of CAUTIs, reflecting its ability to persist on invasive devices and colonize the urinary tract in patients with prolonged hospitalization or underlying comorbidities. Similarly, bloodstream infections identified in this study align with those documented in critically ill patients, in whom *P. aeruginosa* is a clinically relevant agent in the progression toward bacteremia, especially in the context of extended hospital stays and the use of intravascular devices [48,49]. Although the number of SSI-derived *P. aeruginosa* isolates was low, these cases remain clinically relevant because *P. aeruginosa*–associated surgical infections are frequently accompanied by increased morbidity and prolonged recovery [50].

The phenotypic resistance profiles observed demonstrated high levels of resistance to multiple antimicrobial families, particularly fluoroquinolones (63%) and carbapenems (55%), values comparable to those reported in previous studies that have reported elevated resistance rates to both classes [51]. These findings reinforce WHO’s designation of *P. aeruginosa* as a pathogen of difficult therapeutic management, particularly in hospital environments where carbapenems remain key components of antimicrobial therapy [3]. The detection of MDR and XDR phenotypes in our population, although comparatively limited, represents an important epidemiological warning. Similar increased in MDR and XDR *P. aeruginosa* have been documented elsewhere, including studies from Spain and global 20-year surveillance analyses reporting MDR rates of 21.8% and XDR rates of 15.2% [52,53]. At HJM, the presence of MDR and XDR phenotypes, although comparatively limited, represents an epidemiological alert given their therapeutic constraints and association with severe clinical outcomes.

Given the increasing antimicrobial resistance observed in recent years, several therapeutic strategies have been proposed, including monotherapy with new β-lactam/β-lactamase inhibitor combinations (ceftolozane–tazobactam, ceftazidime–avibactam, or imipenem–relebactam). However, these options are often not available, leading to the use of cefiderocol as an alternative [54]. The preserved susceptibility to colistin in our isolates highlights its role as a rescue antibiotic, although its clinical use must be carefully evaluated due to its nephrotoxic and hepatotoxic potential [55].

The detection of the *bla_VIM_* gene in six isolates confirms the circulation of metallo-β-lactamases in HJM, consistent with previous findings that identified this carbapenemase as the predominant enzyme in *P. aeruginosa* [12,31]. The presence of *bla_VIM_* is particularly relevant given its ability to hydrolyze most β-lactams, including carbapenems, and its association with hospital outbreaks and high mortality rates [56]. However, the absence of carbapenemases in some imipenem-resistant strains suggests the involvement of alternative mechanisms.

Regarding the mechanism associated with *oprD* mutations, the analysis revealed recurrent substitutions in loops 1, 2, and 3—regions critical for imipenem uptake. Mutations in loop 3 were of special interest, as previous studies indicate that this region plays an important role in porin structure and interaction with carbapenems. These mutations can modify pore diameter and surface charge, limiting antibiotic entry and contributing to imipenem resistance [57]. Our findings showed that the main mutations were located in loops 2 and 3, consistent with studies linking these regions to loss of imipenem permeability. Molecular studies have shown that loop 3 acts as a primary recognition site for carbapenems, while loop 2 influences channel selectivity [58].

The substitutions detected in oprD loops show recurrent patterns among imipenem-resistant isolates, supporting a non-random signal of variation. However, hypothesized physicochemical or structural effects (backbone flexibility restriction or hydrophobic packing) remain interpretative hypotheses rather than demonstrated functional consequences, as no structural modelling or functional assays were performed. We acknowledge this as a limitation and emphasize that carbapenem resistance in *P. aeruginosa* is multifactorial, and should not be attributed exclusively to oprD loop substitutions or porin mutations alone, which act as permeability-modulating signals within a broader resistome context [59,60]. Nonetheless, confirmation of this interpretation will require experimental structural validation.

The high frequency of substitutions such as L170F and T115K suggests adaptive changes driven by antimicrobial pressure in the hospital environment, consistent with experimental models where prolonged exposure to carbapenems results in *oprD* deletions or point mutations [57]. Likewise, Ocampo-Sosa et al. [61] reported that the most frequent mutations occur in loop 3, in agreement with our results (Table 2). Importantly, while imipenem resistance within the imipenem-resistant subset was addressed through targeted carbapenem-focused screening (*bla* gene detection and OprD loop substitutions), the broader MDR/XDR phenotypes observed across the study set may still involve additional genetic determinants that were not captured by the limited PCR panel or by loop-level porin analysis. This is consistent with the findings of Cortés-Lara et al. [62], who emphasized the need to evaluate the full resistome to better understand the diversity of resistance drivers in *P. aeruginosa*. Accordingly, although whole-genome sequencing was not performed due to the targeted design of this study, it represents a valuable future approach to comprehensively resolve these additional mechanisms and strengthen genotype–phenotype correlations. Unlike recent WGS-based studies aimed at comprehensive resistome profiling, this work employed a targeted candidate-gene strategy to address institution-specific carbapenem resistance mechanisms relevant to routine surveillance.

In recent years, comprehensive genomic analyses have significantly advanced this field. Biggel et al. [63], for example, evaluated complete genomes of *P. aeruginosa* to identify functional loss of OprD from sequencing data. This in silico approach provides a standardized, robust method for correlating genotypes with carbapenem resistance phenotypes. Such advances underscore the importance of incorporating whole-genome sequencing in future research to fully elucidate the resistome of *P. aeruginosa*. Overall, integrating genomic approaches will be essential to fully elucidate resistance mechanisms and inform infection control strategies.

This study provides relevant local insights; however, some limitations should be considered. As a single-center investigation at HJM, the sample size of 63 isolates (including 27 imipenem-resistant strains analyzed for *oprD*) supports institutional surveillance but prevents national generalization. Molecular screening was limited to the main carbapenemases circulating locally *(bla_VIM_*, *bla_NDM_*, *bla_IMP_*, *bla_KPC_*) and does not capture the full resistome. Additionally, loop substitutions in OprD (L1–L3) were identified without experimental structural or functional validation, and whole-genome sequencing was not performed, limiting detection of other resistance determinants. We emphasize that absence of other genes does not exclude alternative resistance mechanisms. We clarify that oprD loop substitutions are permeability-modulating changes, not the sole cause of all carbapenem-resistant phenotypes, which are multifactorial. Imipenem-sensitive isolates were not included in the oprD loop (L1–L3) comparison; therefore, substitutions are interpreted as permeability-modulating signals, but we cannot fully exclude natural polymorphisms. Functional assays or WGS-based PorinPredict analyses may strengthen species-level genotype–phenotype specificity in future studies.

The high frequency of certain OprD loop 2 and 3 substitutions, particularly S103T, T115K, L170F, G186P, T189V, and E203Q, observed in the isolates analyzed may suggest the circulation of closely related strains or the repeated selection of similar adaptive variants under local antimicrobial pressure. Prospective studies incorporating genomic typing and phylogenetic analyses will be required to formally assess clonal relationships and transmission dynamics within the institution.

## 5. Conclusions

Our results show that *P. aeruginosa* remains a highly relevant pathogen at HJM, particularly in the context of HAIs such as VAP and CAUTI. The integration of epidemiological findings, phenotypic resistance profiles, and molecular analyses revealed that carbapenem resistance in the strains studied did not stem from a single mechanism, but rather from the convergence of multiple adaptive strategies. Among these, the presence of the *bla_VIM_* carbapenemase and the structural alterations detected in loops 1, 2, and 3 of the OprD porin—regions that are critical for imipenem entry—stand out. The presence of MDR and XDR phenotypes underscores the importance of maintaining strict and continuous microbiological surveillance, especially in areas where antibiotic pressure is high. These findings help clarify the predominant resistance mechanisms circulating within the local *P. aeruginosa* population and provide a solid foundation for guiding effective therapeutic decisions.

## Figures and Tables

**Table 1:** Origin of *Pseudomonas aeruginosa* isolates of this study.

HAI/Isolation Source	Year/*n* (%)		Sex/*n* (%)	
	2021	2022	Male	Female
^a^ CAUTI/urine	8 (21.6)	6 (23.1)	9 (25.7)	5 (17.9)
^b^ VAP/lung	22 (59.5)	15 (57.7)	21 (60.0)	16 (57.1)
^c^ CLABSI/blood	5 (13.4)	3 (11.5)	3 (8.6)	5 (17.9)
^d^ SSI/wound	2 (5.4)	2 (7.7)	2 (5.7)	2 (7.1)
Total	37 (100)	26 (100)	35 (100)	28 (100)

^a^ Catheter-Associated Urinary Tract Infection, ^b^ Ventilator-Associated Pneumonia, ^c^ Central Line–Associated Bloodstream Infection, ^d^ Surgical Site Infection.

**Table 2:** Summary of amino acid substitutions detected in loops L1, L2, and L3 of the OprD porin among *Pseudomonas aeruginosa* isolates, including their positions and frequency of occurrence.

Loop	Position	Amino Acid Change	Frequency *n* (%)
1	43	D → N	15 (55.6)
1	57	S → E	15 (55.6)
1	59	S → R	15 (55.6)
2	103	S → T	21 (77.8)
2	114	G → S	1 (3.7)
2	115	T → K	21 (77.8)
3	170	L → F	21 (77.8)
3	185	Q → E	20 (68.7)
3	186	G → P	21 (77.8)
3	189	T → V	21 (77.8)
3	190	K → N	2 (7.1)
3	192	S → A	2 (7.1)
3	193	R → V	2 (7.1)
3	194	G → S	2 (7.1)
3	195	E → S	2 (7.1)
3	196	L → T	2 (7.1)
3	197	Y → P	2 (7.1)
3	198	A → P	2 (7.1)
3	200	Y → P	2 (7.1)
3	202	G → R	2 (7.1)
3	203	E → Q	17 (63)

## Data Availability

Bello-López, Juan Manuel (2025), “Genetic Determinants Linked to MDR/XDR in *Pseudomonas aeruginosa* Strains from a Mexican Tertiary Hospital in Mexico”, Mendeley Data, V1, doi: 10.17632/czmj42xy6r.1 (https://data.mendeley.com/drafts/czmj42xy6r (accessed on 10 September 2025)).
